# The influence of personality on memory self-report among black and white older adults

**DOI:** 10.1371/journal.pone.0219712

**Published:** 2019-07-15

**Authors:** Nikki L. Hill, Jacqueline Mogle, Sakshi Bhargava, Tyler Reed Bell, Rachel K. Wion

**Affiliations:** 1 College of Nursing, The Pennsylvania State University, University Park, Pennsylvania, United States of America; 2 College of Health & Human Development, Edna Bennett Pierce Prevention Research Center, The Pennsylvania State University, University Park, Pennsylvania, United States of America; Fordham University, UNITED STATES

## Abstract

Self-reported memory problems are often the first indicator of cognitive decline; however, they are inconsistently associated with objective memory performance and are known to be influenced by individual factors, such as personality. The current study examined the relationships between personality traits and self-reported memory problems in cognitively intact older adults, and whether these associations differ across Black and White older adults. Data were collected annually via in-person comprehensive medical and neuropsychological examinations as part of the Einstein Aging Study. Community-dwelling older adults in an urban, multi-ethnic area of New York City were interviewed. The current study included a total of 425 older adults (*M*_age_ = 76.68, *SD* = 4.72, 62.59% female; 72.00% White). Multilevel modeling tested the associations of neuroticism, conscientiousness, extraversion, openness, and agreeableness with self-reported memory problems. Results showed that neuroticism was positively related to frequency of memory problems and perceived ten-year memory decline only when other personality traits were not accounted for. Extraversion was negatively related to frequency of memory problems and perceived ten-year decline for both White and Black participants. However, conscientiousness was negatively related to perceived ten-year decline for Black participants only. Our findings highlight the importance of examining the association of all five personality traits with self-reported memory problems, as well as examining whether these associations differ for participants from different race/ethnicities.

## Introduction

By 2060, the number of older adults in the United States will increase by over 20%, highlighting healthy aging as a public health priority [1]. However, one in four older adults experiences memory problems that can impede healthy aging [2]. Self-reported memory problems, even in the absence of clinically identifiable cognitive deficit, are associated with disruptions in daily activities, increased functional limitations, elevated injury risk over time, depressive symptoms, and a higher risk for incipient cognitive decline and dementia [3–6]. Deciphering the factors that influence older adults’ reports of memory problems would improve early identification of functional and cognitive problems as well as inform intervention efforts to facilitate promotion of healthy aging.

When it comes to *self*-reported memory, *individual* characteristics play explanatory roles. Often, reports of memory problems cannot be corroborated by cognitive testing [7] and instead may be influenced by demographic and other individual differences as well as affective symptoms. In cross-sectional studies, self-reported memory problems differ by age and sex [8–12]. However, further investigation is needed, particularly with regard to racial differences, as these are rarely studied and results are inconsistent. For example, Luchetti, Terracciano, Stephan, and Sutin [13] found that Black older adults endorsed poorer general ratings of current memory than Whites even after controlling for socioeconomic and health disparities. However, Abner, Kryscio, Caban-Holt, and Schmitt [14] found that Black older adults were less likely to complain of worsening memory problems compared with White older adults. Additionally, while demographic characteristics might identify portions of the population with higher reported memory problems, they cannot fully explain why variation occurs within subgroups. Affective symptoms, particularly depressive symptoms, commonly co-occur with self-reported memory problems [15]. Depression can include cognitive symptoms in addition to negative affect, and memory problems as a presenting feature may be more common among older adults [16,17]. Indeed, in clinical settings, memory complaints uncorroborated by objective testing are often attributed to depression, and one of the most commonly used depression screening tools for older adults, the Geriatric Depression Scale (GDS), includes the item: “Do you feel you have more memory problems than most?” [18] Another potential explanatory indicator that varies across demographic characteristics, and has been implicated in other health-related outcomes, is personality [19–21].

The Five Factor Model (FFM) of personality (neuroticism, extraversion, openness, agreeableness, and conscientiousness) is commonly applied for understanding relationships between personality traits and aging-related outcomes [22,23], including self-reported memory problems. For example, higher neuroticism, which includes tendencies to anticipate and experience distress, is consistently linked with higher reports of memory problems above demographics [13,24–26]. Conversely, both higher conscientiousness (more likely to be responsible, structured, and hardworking; [27]) and higher extraversion (more likely to be genial, social, and expressive; [27]) have been associated with fewer self-reported memory problems in older adults [13]; findings on other FFM traits are decidedly mixed [13,28,29].

Among the FFM traits, neuroticism has been the most powerful predictor of self-reported memory problems in previous research, accounting for nearly one-fifth of the differences between individuals–much greater than variance explained by other FFM traits or psychological states [24,29]. Although neuroticism is consistently associated with cognitive, emotional, and physical health outcomes [30], consideration of the full FFM is crucial to our understanding of personality’s influence on health outcomes [31]. Research suggests that personality factors and their functions, like that of neuroticism, may differ somewhat across certain ethnic and racial makeups [32]. Thus, there is a need to evaluate whether personality independently accounts for reports of memory problems (suggested by Luchetti et al. [13]), or through a unique contribution of personality traits within demographic subgroups, such as between Black and White older adults (i.e., moderating effects). Such clarification may better discern older adults at risk for later cognitive decline and other important outcomes.

The purpose of this study was to examine the influence of personality traits on memory self-report among older adults without cognitive impairment, and to determine whether these associations differ by race, specifically between Blacks and Whites. These relationships were tested using the FFM traits (neuroticism, extraversion, openness, agreeableness, conscientiousness) to investigate whether personality traits influenced responses to three memory self-report items (frequency of memory problems, perceived one-year decline in memory, and perceived ten-year decline in memory).

## Methods

### Participants

Data were drawn from the Einstein Aging Study (EAS), a longitudinal cohort study examining cognitive aging and dementia among community-dwelling older adults (70+ years) in an urban, multi-ethnic area of New York City. EAS data were collected annually via in-person comprehensive medical and neuropsychological examinations. Study participants completed written, informed consent upon their initial clinic visit [33]. The study protocol was approved by the Albert Einstein College of Medicine Institutional Review Board. Full study details are described elsewhere [33]. Personality measures were added in 2005 and therefore, of the 2,074 EAS participants who completed self-reports of memory, only 730 participants completed personality measures. Further, of these 730 participants, 271 (37.12%) were classified as having either amnestic mild cognitive impairment (MCI), non-amnestic MCI, or dementia, and thus excluded from the current study. Participants were asked to self-identify their primary ethnic group as Caucasian, African American, Hispanic (Black or White), Asian, or other. Individuals who identified as Hispanic (Black or White), Asian, or Other (7.21%) were also excluded as there were insufficient numbers of participants to appropriately model these groups. One additional individual was excluded as their MCI status was not available. Therefore, the current study included 425 participants (72.00% White, 62.59% female) who were at least 70 years old (*M*_age_ = 76.68, *SD* = 4.72) and had no clinical diagnosis of MCI or dementia at any point throughout the study period. Up to 11 years of data were included for each participant (*M*_years_ = 4.00, *SD* = 2.69). At baseline, participants had an average of 14.91 years of education (*SD* = 3.08). Participants were also asked to report their current income in broad categories (less than $15,000, between $15,001 and $30,000, greater than $30,000). A total of 9.87% had an annual income below $15,000 (i.e., lived below poverty level); 33.67% of the participants’ annual income was between $15,001 and $30,000 (i.e., lived at poverty level to up to two times above poverty level); 56.56% of the participants had an annual income above $30,000 (i.e., lived more than two times above poverty level).

### Measures

#### Memory self-report

Three measures of self-reported memory were administered at each annual visit. Frequency of memory problems was assessed with the item, “*In the past year*, *how often did you have trouble remembering things*?,” with response options on a four-point scale: 1 = never, 2 = rarely, 3 = sometimes, 4 = frequently. Perceived one-year decline in memory was assessed with the item, *“Compared with one year ago*, *do you have trouble remembering things more often*, *less often*, *or about the same*?*”* Perceived ten-year decline in memory was assessed with the item, “*Compared with ten years ago*, *do you have trouble remembering things more often*, *less often*, *or about the same*?” For both items assessing perceived decline in memory, options were less often, about the same, and more often. Responses indicating problems occurred less often were infrequent (2.13%– 4.50%) and were re-coded to: 0 = less often/about the same and 1 = more often.

#### Personality

The 50-item International Personality Item Pool (IPIP) questionnaire [34] was used to measure the FFM personality traits: neuroticism (e.g., *“I often feel blue”*), conscientiousness (e.g., *“I pay attention to details”*), extraversion (e.g., *“I feel comfortable around people”*), agreeableness (e.g., *“I accept people as they are”*), and openness (e.g., *“I enjoy hearing new ideas”*). Participants responded to 10 items for each personality trait, with response options on a five-point scale: 1 = very inaccurate, 2 = moderately inaccurate, 3 = neither inaccurate nor accurate, 4 = moderately accurate, 5 = very accurate). Negatively worded items were reverse scored, and a total score was created for each personality trait with higher scores indicating higher neuroticism, conscientiousness, extraversion, agreeableness, and openness.

#### Depressive symptoms

The 15-item Geriatric Depression Scale (GDS-15; [18]) was used to measure depressive symptoms at each annual visit. Participants responded “yes” or “no” to a series of statements, based on how they felt over the past week. Due to the overlap with self-reported memory, the GDS was adjusted for the current analyses to remove the item “*Do you feel you have more problems with memory than most*?” [35,36] Therefore, scores ranged from 0–14 with higher scores indicating more depressive symptoms. The GDS-15 has been found to be reliable in older adults (*α* = 0.729; [37]), and is significantly associated with measures of depressed mood, life satisfaction, and suicidal ideation, demonstrating construct validity [37].

### Analysis

Prior to examining the proposed research questions, descriptive analyses were performed to investigate if all variables of interest were normally distributed. Mean differences in age, education, depressive symptoms, frequency of memory problems, one-year and ten-year memory decline, and personality traits were examined by race and sex. Next, inter-correlations were examined among the key study variables. Correlations with categorical variables were calculated using the Kendall Tau correction (e.g., sex).

Multilevel modeling (MLM) was performed in SAS (v. 9.4) to examine average associations among older adults’ personality traits and self-reported memory (i.e., frequency of memory problems, perceived one-year memory decline, and perceived ten-year memory decline). Using MLM for this type of analysis allows us to control for the influence of time while also accounting for missing data due to unequal follow up among participants. MLM uses maximum likelihood estimation techniques where all participants can be included in analyses regardless of missing years of follow up data [38]. Frequency of memory problems was treated as a continuous outcome and modeled using SAS proc mixed. Perceived one- and ten-year decline were binary variables (0 = less often/about the same and 1 = more often) and were modeled using SAS proc glimmix using a binary distribution with a logit link. First, empty models examined intraclass correlations to determine the proportion of variance in perceived frequency of memory problems and memory decline that could be explained by individual differences in our sample. For the first set of substantive analyses, models examined the association of neuroticism with self-reported memory problems. Next, the simultaneous association of all five personality traits (i.e., neuroticism, conscientiousness, extraversion, agreeableness, and openness) with self-reported memory problems was examined. Last, to examine whether the association of personality traits with the three types of memory self-report significantly differed for Blacks and Whites, interactions of race with personality traits were added to the model. Final models included only significant interaction terms. Participants’ age, sex (0 = female; 1 = male), race (0 = Black; 1 = White), education, income level, depressive symptoms, and time in study were included as covariates in all models. Personality variables were included as between-person variables and were grand-mean centered. Baseline depressive symptoms were grand-mean centered. Additionally, participants’ age and education were grand-mean centered, and income was dummy coded with the category $15,001 and $30,000 as the reference category. Effect sizes included odds ratios (ORs) for binary outcomes and pseudo *R*^2^s for continuous outcomes [39].

As only a subsample of EAS participants completed the personality measures, we tested for differences in those with and without personality data. Significant differences were observed in age, education level, and depressive symptoms. Participants who completed personality measures were younger [M_completed_ = 76.68, S.D. = 4.72; M_didnotcomplete_ = 78.44, S.D. = 5.17; t (929.5) = 6.02, *p* < .001], had more years of education [M_complete_ = 14.91, S.D. = 3.08; M_didnotcomplete_ = 12.66, S.D. = 3.63; t (990.60)] = -11.43, *p* < .001], and reported fewer depressive symptoms [M_complete_ = 1.58, S.D. = 1.75; N_didnotcomplete_ = 2.59, S.D. = 2.49; t (985.36) = 7.44, *p* < .001] than their counterparts. No significant race, sex, or memory self-report differences were observed in participants who completed personality measures compared to those who did not.

## Results

### Baseline comparisons

Participants’ age, education level, and self-reported memory problem frequency differed by race. White participants (*M* = 77.150, *SD* = 4.972) were older than Black participants (*M* = 75.479, *SD* = 3.766; *t*(282.02) = -3.74, *p* < .001). Also, White participants (*M* = 15.134, *SD* = 3.126) reported more years of education than Black participants (*M* = 14.336, *SD* = 2.903; *t*(423) = -2.41, *p* = .016). In addition, White participants reported more frequent memory problems (*M* = 2.673, *SD* = 0.666) than Black participants (*M* = 2.513, *SD* = 0.754; *t*(410) = -2.11, *p* = .035). Personality traits did not vary by race.

Sex differences were observed in self-reported memory problem frequency such that females reported more frequent memory problems (*M* = 2.716, *SD* = 0.650) than males (*M* = 2.484, *SD* = 0.742; *t*(410) = 3.33, *p* < .01). Additionally, sex differences were observed in some personality traits such that females had higher levels of neuroticism and agreeableness (neuroticism: *M* = 21.060, *SD* = 6.337; agreeableness: *M* = 41.263, *SD* = 4.873) than males (neuroticism: *M* = 19.830, *SD* = 5.747; *t*(423) = 2.00, *p* = .046; agreeableness: *M* = 40.132, *SD* = 5.451; *t*(423) = 2.21, *p* = .027).

### Descriptive statistics

Intercorrelations among key study variables are presented in Table 1.

**Table 1:** Inter-correlations among key study variables.

	1	2	3	4	5	6	7	8	9	10	11	12	M (SD)
1. Age	-												76.68 (4.72)
2. Education	-.07	-											14.91 (3.08)
3. Income	-.09*	.20***	-										2.47 (0.67)
4. Depressive Symptoms	.04	-.13**	-.06	-									1.58 (1.75)
5. Frequency of Memory Problems	-.03	.04	-.01	.05	-								2.63 (0.69)
6. Perceived One-year Decline	-.06	.06	.04	.26***	.22***	-							0.14 (0.35)
7. Perceived Ten-year Decline	.04	.04	.04	.01*	.31***	.29***	-						0.61 (0.49)
8. Neuroticism	.07	-.15**	-.07^+^	.49***	.16***	.14**	.10*	-					20.60 (6.14)
9. Conscientiousness	-.03	.14**	.07^+^	-.33***	-.18***	-.10*	-0.05	-.41**	-				38.53(6.37)
10. Extraversion	-.02	.10*	.06	-.24***	-.12*	-.16***	-.10**	-.26***	.37***	-			34.13 (6.40)
11. Agreeableness	.14**	-.03	.004	-.16**	-.10*	-.11**	0.01	-.45***	.270***	.22***	-		40.84 (5.12)
12. Openness	-.01	.50***	.12**	-.10*	-.02	0.03	0.02	-.18***	.23***	.30***	.19***	-	37.37 (6.46)

***p ≤ .001.

**p ≤ .01.

*p ≤ .05.

^+^*p* < .10

### Multilevel models

#### Intraclass correlation coefficients (ICC)

Prior to examining whether personality traits were related to self-reported memory over time, ICCs were examined. The ICCs showed that 50.2% of the variation in self-reported frequency of memory problems, 49.5% of the variation in perceived one-year memory decline, and 56.1% of the variation in perceived ten-year memory decline were accounted for by individual differences in our sample.

#### Substantive models

Substantive models examined: 1) associations of neuroticism with self-reported memory, 2) associations of all five personality traits with self-reported memory, and 3) whether associations of personality traits with self-reported memory differed for Black and White older adults. Participants’ age, education level, race, sex, income, and depressive symptoms were accounted for in all models. Findings for the association of personality traits with self-reported memory and multigroup analyses are provided below by type of memory self-report.

#### Frequency of memory problems

After accounting for covariates, results showed that neuroticism was significantly associated with frequency of memory problems (*b* = 0.016, *SE* = .005, *p* < .01, pseudo *R*^2^ = 1.4%), such that participants with higher neuroticism perceived more frequent memory problems (see Table 2, Model 1a). However, when other personality traits were included in the model (see Table 2, Model 1b), neuroticism was no longer significantly related to memory problem frequency. Instead, participants’ conscientiousness and extraversion were significantly related to memory problem frequency (*b*_conscientiousness_ = -.012, *SE* = .005, *p* = .017, pseudo *R*^2^ = 2.7%; *b*_extraversion_ = -.009, *SE* = .005, *p* = .048, pseudo *R*^2^ = 3.1%), such that older adults with higher conscientiousness and extraversion reported a lower frequency of memory problems compared to their counterparts. Race did not moderate the association between personality traits and self-reported frequency of memory problems.

**Table 2:** Estimates from multilevel modeling examining association of personality with self-reported memory.

	Frequency of Memory Problems		Perceived One-Year Memory Decline		Perceived Ten-Year Memory Decline	
	Model 1a b (S.E.)	Model 1b b (S.E.)	Model 2a OR (95% CI)	Model 2b OR (95% CI)	Model 3a OR (95% CI)	Model 3b OR (95% CI)
Time	0.010* (0.005)	0.010* (0.005)	1.080** (1.020–1.142)	1.078** (1.019–1.141)	1.102*** (1.041–1.167)	1.102** (1.041–1.166)
Age	-0.015* (0.006)	-0.014* (0.006)	0.927** (0.878–0.979)	0.929** (0.879–0.981)	0.956 (0.906–1.009)	0.953^+^ (0.904–1.006)
Education	0.008 (0.009)	0.008 (0.011)	1.077^+^ (0.991–0.171)	1.086^+^ (0.985–1.197)	1.013 (0.930–1.105)	1.017 (0.923–1.122)
Black (ref = White)	-0.075 (0.065)	-0.091 (0.065)	0.667 (0.367–1.213)	0.633 (0.345–1.162)	0.697 (0.384–1.262)	0.709 (0.392–1.284)
Female (ref = Male)	0.132* (0.059)	0.140* (0.059)	1.201 (0.714–2.019)	1.226 (0.725–2.072)	1.682^+^ (0.980–2.886)	1.519 (0.885–2.608)
Income < $15,000 (ref = $15,001 − $30,000)	-0.166 (0.104)	-0.149 (0.103)	0.639 (0.240–1.703)	0.689 (0.260–1.829)	0.640 (0.250–1.639)	0.633 (0.250–1.604)
Income > $30,000 (ref = $15,001 − $30,000)	-0.018 (0.062)	-0.013 (0.062)	1.519 (0.872–2.644)	1.542 (0.888–2.676)	1.651^+^ (0.935–2.915)	1.689^+^ (0.964–2.960)
Depressive Symptoms	0.017 (0.018)	0.005 (0.018)	1.318*** (1.129–1.538)	1.263** (1.081–1.476)	1.285** (1.078–1.531)	1.235* (1.035–1.474)
Neuroticism	0.016** (0.005)	0.008 (0.006)	1.047* (1.002–1.094)	1.026 (0.974–1.080)	1.032 (0.984–1.082)	1.035 (0.981–1.093)
Conscientiousness	-	-0.012** (0.005)	-	0.967 (0.927–1.009)	-	0.973 (0.927–1.022)
Extraversion	-	-0.009* (0.005)	-	0.960^+^ (0.921–1.001)	-	0.955* (0.914–0.998)
Agreeableness	-	-0.004 (0.006)	-	0.997 (0.944–1.052)	-	1.033 (0.976–1.093)
Openness	-	0.003 (0.005)	-	1.002 (0.958–1.049)	-	1.008 (0.963–1.056)
Conscientiousness*Race	-	-	-	-	-	0.909* (0.834–0.992)

*Note*. Only significant interaction terms are shown in the table.

***p < .001.

**p < .01.

*p < .05.

^+^*p* < .10.

#### Perceived one-year memory decline

After accounting for covariates, results showed that neuroticism was significantly related to perceived one-year memory decline (OR: 1.047; 95% CI: 1.002–1.094), such that older adults with higher neuroticism were more likely to report a one-year memory decline compared to their counterparts (see Table 2, Model 2a). However, this relationship did not remain significant after other personality traits were added to the model (see Table 2, Model 2b). No personality trait was significantly related to one-year memory decline.

#### Perceived ten-year memory decline

After accounting for covariates, results showed that neuroticism was not significantly related to perceived ten-year memory decline (OR: 1.032; 95% CI: 0.984–1.082; see Table 2, Model 3a). The model with all personality traits showed that extraversion was related to perceived ten-year memory decline (OR: 0.955: 95% CI: 0.914–0.998), such that older adults higher in extraversion were less likely to perceive a ten-year memory decline than those lower in neuroticism (see Table 2, Model 3b). Race moderated the association of conscientiousness with perceived ten-year memory decline (OR: 0.909; 95% CI: 0.834–0.992), such that Black older adults lower in conscientiousness were more likely to report a ten-year memory decline than Black older adults higher in conscientiousness. Conscientiousness was not related to White older adults’ reports of ten-year memory decline. Other associations between personality traits and perceived ten-year memory decline in the full model did not differ for White and Black older adults (all ps > 0.21).

## Discussion

The purpose of the current study was to examine the influence of FFM personality traits (neuroticism, extraversion, openness, agreeableness, conscientiousness) on self-reported memory problems among cognitively intact older adults and whether these relationships were moderated by race. Consistent with some previous research [13,24,26], neuroticism predicted self-reported frequency of memory problems and perceived recent (one-year) decline when neuroticism was the only personality variable in the model. However, when the remaining four personality traits were included, neuroticism was no longer a significant predictor of memory self-report in this sample. Although most relationships were equivalent across the two racial groups in this sample, we found that conscientiousness impacted reports of perceived ten-year memory decline in Black older adults but not White. These results suggest that the influence of personality on self-reported memory may not be consistent across racial groups and supports the need for further research into racial and ethnic differences in factors influencing memory self-report.

Our findings regarding neuroticism are contrary to previous research that supports a relatively ubiquitous influence such that individuals higher in neuroticism tend to report more memory problems. There are several possible reasons for this difference. First, our participants were older and had lower neuroticism scores, on average, compared to samples in previous work; aging is significantly related to decline in neuroticism [40]. Therefore, our findings might reflect a dampening of neuroticism’s impact compared to the effects seen at younger ages [24]. Second, most studies examine neuroticism alone, which might mask the importance of other traits on self-reported memory (i.e., significant effects might indirectly derive from associations with other trait tendencies). Once all FFM traits were considered, extraversion and conscientiousness were significantly related to reported frequency of memory problems and perceived ten-year decline. Our results suggest a need to carefully consider how personality influences memory self-reports: focusing on neuroticism alone may oversimplify more complex relationships.

In this sample, higher extraversion was associated with lower reported frequency of memory problems as well as less perceived ten-year decline. Individuals higher in extraversion are excitement-seeking, assertive, and cheerful. According to Eysenck’s theory of extraversion [41], this “liveliness” promotes cortical arousal and engagement for cognitive tasks. As processing capacity deteriorates with age, the preference for engagement may require additional cognitive effort needed for normal memory recall [42,43]. Alternatively, seeking exciting, complex social situations keeps one involved in cognitively rich activities, thereby potentially enhancing memory skills and buffering against noticeable decline. Both of these reasons may explain why individuals with higher extraversion scores are less likely to experience cognitive decline compared to their peers [13]. Moreover, higher extraversion is also associated with greater global self-efficacy, which could improve one’s confidence about their own memory (and therefore influence memory self-report).

Similar to extraversion, we found that higher conscientiousness was associated with a lower reported frequency of memory problems. Older adults higher in conscientiousness may exert greater cognitive effort, leading to improved performance on memory tasks [13,44]. Compared to extraversion, this exertion is motivated by a need to perform well rather than higher engagement. Such motivations can inspire people to approach memory challenges with careful, goal-focused mindsets, leading older adults with higher conscientiousness to employ memory strategies and compensate for near-lapses [45]. As with higher extraversion, older adults with higher conscientiousness report higher self-efficacy, particularly when it comes to memory [46], which might reduce perceived frequency of memory problems.

Interestingly, we found that higher conscientiousness was associated with less perceived ten-year decline among Black older adults only. Although further exploration is needed, this may be partially explained by health disparities affecting Black older adults including more chronic illnesses [47] and pain [48]—factors that contribute to self-reported memory problems [49,50]. Since higher conscientiousness increases engagement in positive health behaviors, it could uniquely improve well-being and associated views about memory [51]. Future work should explore how conscientiousness affects differential mechanisms behind long-standing memory perceptions in Black and White older adults.

This study had several limitations to consider. First, the sample was limited to those living in a large urban area in the northeast. However, this did allow for a multi-ethnic sampling frame. Second, the FFM is a descriptive model not founded in theory but rather constructed by factor analyses [52]. Moreover, there is no true theory to explain why personality traits cluster together (although concepts like self-control and emotional regulation might help our understanding). We could consider other personality models like the Zuckerman and Kuhlman’s Alternative Five model [53] or the HEXACO model of personality [54]. However, the FFM is a powerful model that predicts a host of outcomes reliably and universally in numerous studies, including those investigated in older adult populations [55–57]. Additionally, we were unable to include data for races or ethnicities other than Blacks and Whites due to low numbers of Hispanics, Asians, and other races in the EAS dataset.

## Conclusion

Certain personality traits (i.e., higher neuroticism, lower conscientiousness), as well as self-reported memory problems in the absence of objective cognitive deficits, have been found to increase the risk for cognitive impairment in older adults [5,58,59]. Our findings that older adults with higher conscientiousness and extraversion reported less frequent memory problems, and that these effects were stronger than the potential influence of neuroticism, hold important implications for refining our understanding of self-reported memory. Although personality, particularly neuroticism, has been implicated as a contributor to older adults’ memory self-reports in multiple studies [13,24–26], to our knowledge this is the first investigation of the moderation of race on these relationships. Given the critical need to better understand, and respond to, health disparities that influence cognitive decline risk, our study provides initial evidence that racial differences in self-reports of memory performance should be further examined.
